# A Microlens Array Grating for Miniature Multi-Channel Spectrometers

**DOI:** 10.3390/s23208381

**Published:** 2023-10-11

**Authors:** Shuonan Shan, Jingwen Li, Peiyuan Liu, Qiaolin Li, Xiaohao Wang, Xinghui Li

**Affiliations:** 1 Shenzhen International Graduate School, Tsinghua University, Shenzhen 518055, China; ssn21@mails.tsinghua.edu.cn (S.S.); jw-li20@mails.tsinghua.edu.cn (J.L.); liu-py19@tsinghua.org.cn (P.L.); wang.xiaohao@sz.tsinghua.edu.cn (X.W.); 2 Tsinghua-Berkeley Shenzhen Institute, Tsinghua University, Shenzhen 518055, China

**Keywords:** microlens array, grating, fabrication, PDMS, soft lithography, miniature multi-channel spectrometer

## Abstract

Most existing multi-channel spectrometers are constructed by physically stacking single-channel spectrometers, resulting in their large size, high weight, and limited number of channels. Therefore, their miniaturization is urgently needed. In this paper, a microlens array grating is designed for miniature multi-channel spectrometers. A transmissive element integrating microlens arrays and gratings, the MLAG, enables simultaneous focusing and dispersion. Using soft lithography, the MLAG was fabricated with a deviation of less than 2.2%. The dimensions are 10 mm × 10 mm × 4 mm with over 2000 available units. The MLAG spectrometer operates in the 400–700 nm wavelength range with a resolution of 6 nm. Additionally, the designed MLAG multi-channel spectrometer is experimentally verified to have independently valid cells that can be used in multichannel spectrometers. The wavelength position repeatability deviation of each cell is about 0.5 nm, and the repeatability of displacement measurements by the chromatic confocal sensor with the designed MLAG multi-channel spectrometer is less than 0.5 μm.

## 1. Introduction

Spectrometers are used to obtain wavelength–light intensity relationships, which have important applications in many research fields, such as the spectral characteristics of different clinical conditions acquired by spectrometers including diagnosis of human eye diseases, monitoring of multiple gaseous air pollutants, chromatic confocal microscopes, and climate monitoring [1,2,3,4,5]. In recent years, spectrometer research has evolved towards broad spectral ranges, miniaturization, and ease of installation [6,7,8]. Meanwhile with the abundance of measurement scenarios, the demand for flat scanning and even three-dimensional information measurements has increased dramatically, which requires multi-channel spectrometers with multi-line processing capabilities, with requirements for measurement efficiency and data processing speed [9,10,11,12]. Thus, some novel methods for multi-spectrometers have been proposed in recent years, such as spectrometer arrays, which can be used to obtain and analyze multi-channel signals [13,14,15,16].

There are already a number of proven and reliable commercial multi-channel spectrometers available. The multi-channel spectrometer made by Ocean Optics has eight channels that can detect wavelengths from 180 to 1100 nm with a resolution of 0.1 nm and which weighs of 7 kg [17]. The multi-channel spectrometer made by HORIBA has 96 channels that can detect wavelengths from 360 to 780 nm with a resolution of less than 3.5 nm [18]. The dimensions and weights of these current products are too big and heavy due to the simple combination of the multi-channel spectrometers. Therefore, some novel products, in which the functions of focusing and dispersing are integrated into a small optical component, are proposed for the miniaturization of spectrometers [19,20]. The channel number is less than 100, which could not meet the requirement of industry manufacturing. Moreover, the multi-channel spectrometers here are constructed by physically stacking single-channel spectrometers, resulting in their large size, high weight, and limited number of channels. So, their miniaturization is urgently needed.

In existing research, there are several studies that propose some innovative multi-channel spectrometers. Silke Traut et al. used photoresist and holography to fabricate a composite structure with a microlens array on one side and a microlens array grating on the other, whose resolution is more than 10 nm [21]. In Tadayuki Hirano’s research, the structure is designed as a blazed grating-air gap-microlens array and is fabricated by physical vapor deposition and electron beam direct writing. The element can resolve color images but with a low resolution [22]. Jun Shi et al. fabricated a microlens array grating structure by hot melting, self-assembly, and replication on the same surface. A resolution of up to 6.9 nm in the wavelength range of 450 nm~650 nm was achieved [23]. However, these fabrication methods require a high level of environmental cleanliness and materials. The lack of cleanliness during the production process and the curing shrinkage effect of the material can have a negative impact on the results [24,25].

In this paper, a microlens array grating (MLAG) structure [26,27,28], which has a microlens array on one side and a grating on the other side, was designed, fabricated, and verified [29,30]. Soft lithography was employed to fabricate the MLAG with PDMS [31,32,33,34,35,36]. The surface form of the fabricated component was inspected by AFM for the fabrication quality. The performance of the miniature multi-channel spectrometer with the fabricated microlens array grating was verified by some experiments. Furthermore, the application of this multi-channel spectrometer with the microlens array grating for the confocal sensor array was introduced, which can be developed for line-scanning measurements and/or confocal microscopy.

## 2. Theoretical Analysis

**Figure 1:**
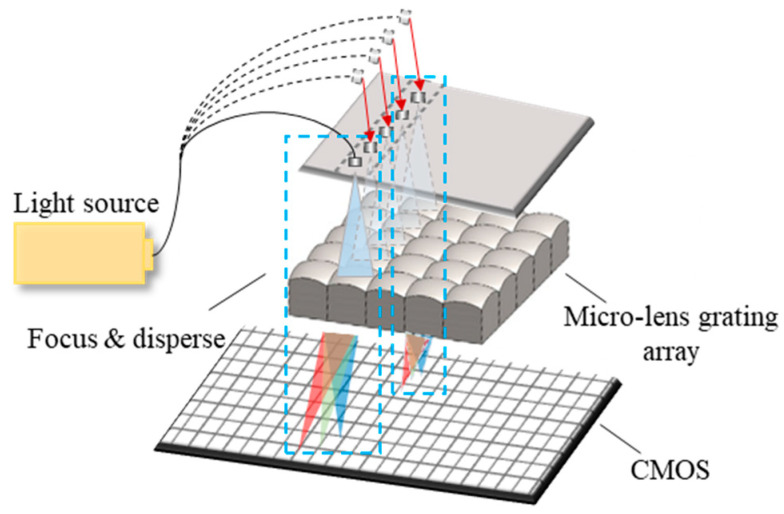
Schematic of a miniature multi-channel spectrometer with the microlens array grating.

Conventionally, the lens and grating are employed in the spectrometer as two different elements for focusing and dispersing, respectively [37,38]. In this paper, the lens and grating are integrated into one component for the miniature spectrometer. As shown in Figure 1, a structure with many microlenses arrayed on one side and a grating on another side of the component surface, which is called microlens array grating, was designed for the multi-channel spectrometer.

### 2.1. Principle of the Microlens Array Grating

**Figure 2:**
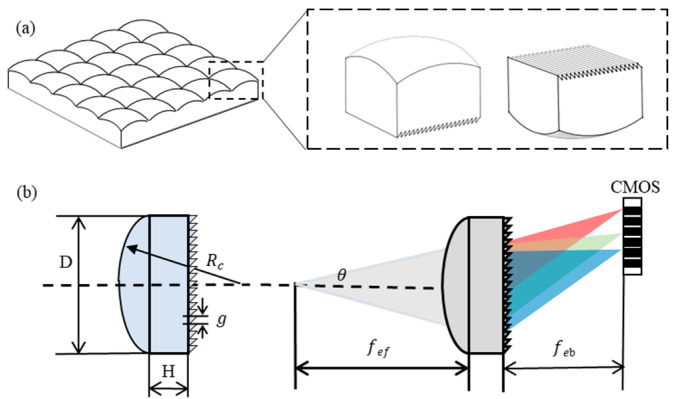
Design of a single unit of the microlens array grating: (**a**) the schematic of a single unit of the microlens array grating; (**b**) the principle of focusing and dispersing by the microlens grating.

As shown in Figure 2a, a miniature multi-channel spectrometer can be realized since only a single unit of the microlens array grating can have the function of focusing and dispersing. A miniaturization of a conventional lens is employed as the microlens for focusing. Considering the complexity of the process and the requirements of the array design, the focusing of the core unit of the spectrometer is realized with a spherical convex lens, whose structure and main parameters are shown in Figure 2b, with *f_ef_* as the effective front focal length, *f_eb_* is the effective back focal distance, $R_{c}$ is the radius of curvature, D is the lens diameter, and H is the unit thickness. A transmission grating, which can periodically modulate the amplitude and phase of the incident light, is employed for diffraction. The grating line density is 600 lines/mm.

### 2.2. Design of the Microlens Array Grating

Designing a microlens array grating (MLAG) spectrometer requires consideration of performance and dimensional goals and ensuring manufacturing feasibility. Following an analysis of research spectrometers, the target specifications were established as follows: physical dimensions below 1 cm × 1 cm × 1 cm, a measurement spectral range of 400–760 nm, and a resolution exceeding 10 nm. According to the fundamental imaging principle, the object distance roughly equals half the size of the spectrometer. Initially, a convex lens with an effective focal length (EFL) of 4 mm was selected to ensure an appropriate size. The grating period was set at 600 lines/mm, favoring a smaller grating period to achieve a larger diffraction angle and enhance optical performance. The preset MLAG parameters included a microlens diameter of 220 μm, microlens focal length of 4 mm, grating line density of 600 lines/mm, and microlens grating thickness (H) of 4 mm.

By utilizing an MLAG as its core component, the spectrometer model can be established with fiber optic arrays serving as the light source input and CMOS photodetectors as the signal reception. Through careful parameter design, simultaneous parallel analysis of various detection channels can be achieved, resulting in an array-type multi-channel micro-spectrometer.

The resolution of the spectrometer is significantly influenced by the pupil diameter. Theoretically, when the aperture diameter is excessively large, the image is magnified, resulting in reduced resolution. Conversely, if the aperture diameter is too small, there is a greater loss of available optical power, leading to reduced resolution as well. Therefore, after careful consideration, a pupil diameter of 10 μm was selected for the spectrometer. When light waves pass through the aperture, diffraction occurs. It is important to note that longer wavelengths result in larger divergence angles. Hence, the maximum divergence angle within the visible range (the maximum wavelength is 760 nm) is as shown in Equation (1) where $\theta_{0}$ is the divergence angle, λ is wavelength D is entrance pupil diameter:

(1) $$\theta_{0}=1.22\frac{\lambda }{D}$$

And the maximum incident half-angle width allowed by the preset microlens parameters is shown in Equation (2) where $\theta_{0}^{\prime }$ is the maximum incident angle.

(2) $$\theta {=\theta }_{0}^{\prime }$$

As a consequence, the spot radius within this range will exceed the maximum limit permissible for the microlens surface element. To mitigate optical power loss and minimize the divergence angle of the light source, a three-piece objective lens set was implemented between the entry pupil and the MLAG. This integration ensures that the exit angle remains at or below 0.0125 rad.

### 2.3. The Simulation of the Designed Microlens Array Grating for the Miniature Multi-Channel Spectrometer

The simulation was carried out using ZEMAX for testing the performance of the spectrometer. For the spectrometer with the proposed microlens array grating, 11 groups of 22 wavelengths ranging from 400 nm to 700 nm were selected. Each group consisted of two wavelengths with similar values. The resolution of the spectrometer could be evaluated based on the relative positions of these two wavelengths in the image-plane spot.

A set of discrete sampling field-of-view points with different positions was established. The simulated fiber core diameter was 105 μm, with seven sampling points at horizontal positions (0, ±0.5 HFOV, ±0.707 HFOV, ±HFOV) and five sampling points at vertical positions (0, ±0.707 HFOV, ±HFOV). Subsequently, the spectrometer system simulation model was created using the lens data. The diffracted spectra from the MLAG, resulting from the passage of light waves through the lens group, were observed to fall on the image plane according to their respective wavelengths.

Table 1 presents the measured resolution of the MLAG spectrometer in each spectral band. The results demonstrate that the system achieved excellent resolution in the 400 nm to 700 nm band, with most positions achieving a resolution of 10 nm or less. In the lower frequency bands, the system achieved an optimal resolution of up to 6 nm.

This performance may not be as favorable compared to commercial spectrometers. However, it is important to note that the primary focus of the MLAG spectrometer is its size reduction and increased processing speed as a multi-channel, array-based miniature spectrometer. Therefore, the loss of resolution is within an acceptable range given these priorities.

## 3. Fabrication and Characterization

### 3.1. Fabrication Process

**Figure 3:**
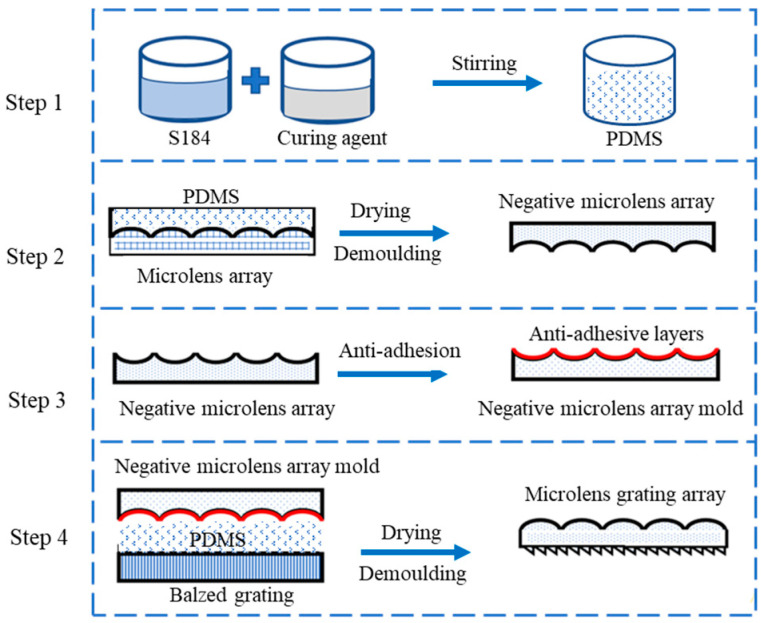
Fabrication process of microlens array grating (MLAG).

Soft lithography technology was chosen to fabricate microlens array gratings in a “sandwich” style. The main body of the microlens array grating consists of an elastic mold, while the grating and microlens array molds were used for printing on the two sides, respectively. It is worth noting that the imprinting of the microlens array involved the use of a negative mold, which was utilized to obtain the desired structure. The overall fabrication process is illustrated in Figure 3. This technique does not rely on expensive lithography equipment, and it enables the shaping of microstructures with nanometer resolution.

Prior to the fabrication process, the imprinting mold was selected according to the designed spectrometer unit. The microlens array was selected from the ML-S220-F4 lens array produced by Shanghai Microlight Technology Co., Shanghai, China. The lens is a square spherical mirror with a focal length of 4 mm, a subunit edge length of 220 μm, and a subunit size of φ16 mm × 2 mm with a size of 12.7 mm × 12.7 mm × 6 mm.

In the first step, PDMS was chosen as the elastic mold material due to its low interfacial separation energy (21.6 dyn/cm), chemical stability, thermal stability, homogeneity, isotropy, and high replication accuracy. The PDMS used was the Sylgard 184 two-component PDMS from Dow Corning, mixed in a ratio of 10:1 with the curing agent. The mixture was thoroughly stirred, and any air bubbles were eliminated using a vacuum to achieve transparency and readiness for use.

**Figure 4:**
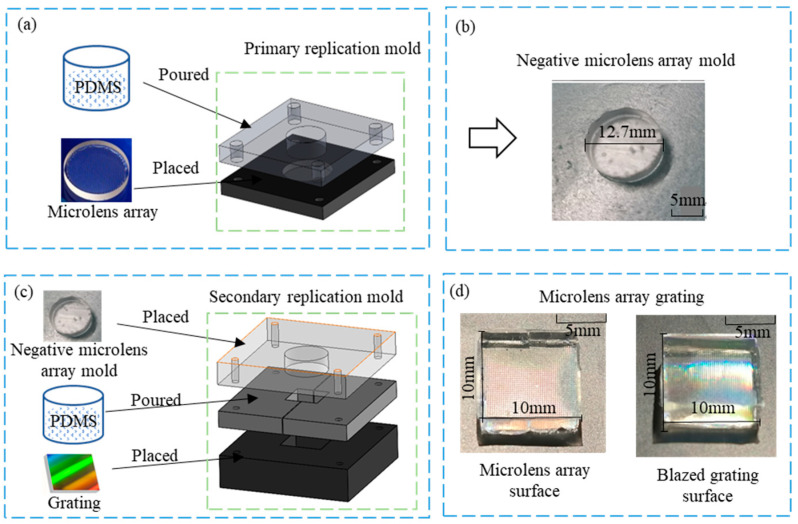
Soft lithography tools and results: (**a**) primary replication process; (**b**) inverted mold of the microlens array; (**c**) secondary replication process; (**d**) both sides of microlens array grating.

The primary replication process was designed to create a negative microlens array mold, as shown in Figure 4a. Special molds were used to pre-mount the microlens arrays, and a bubble-free PDMS mixture was poured slowly over the microlens arrays inside the replica molds. The assembly was then placed in a vacuum-drying oven and baked at 80 °C for 2 h to solidify the PDMS. The negative microlens array was obtained by demolding the solidified PDMS. Since both the negative microlens array and the MLAG are made of PDMS, they tend to adhere to each other. To prevent this, an anti-adhesive coating was applied in Step 3.

Molecular vapor deposition (MVD) was used for the anti-adhesive treatment of the negative microlens array. Two coating materials, Parylene C and Fluorine Nano, were selected. Both coatings had a thickness of 400 nm, which corresponds to approximately 12% of the microlens thickness of 3.32 μm. It can be assumed that the film layer has minimal impact on the microlens morphology, size, and focusing effect. Subsequently, separate tests were conducted on the MLAGs fabricated using the two different coatings.

After the negative microlens array was obtained, it and the grating could be used as molds for “sandwich-type” MLAG fabrication, as shown in Figure 4b. The process is similar to step 2, that is, the PDMS was poured into a special mold, the negative microlens array with anti-stick coating and blazed grating fixed in the mold of the upper and lower layers, and then the whole unit was placed in a vacuum oven, curing at 80 °C for 2 h. Then, the mold was removed, cooled, and demolded to obtain an MLAG with dimensions of 10 mm × 10 mm × 4 mm containing more than 2000 spectrometer units.

Dedicated molds were used in both steps 2 and 4 in order to complete the alignment and fabrication of the two micro-structured graphic planes, the two molds are shown in Figure 4a,b, respectively, and the specific parameters are shown in Table 2 and Table 3.

### 3.2. Characterization of Microlens Array Grating

**Figure 5:**
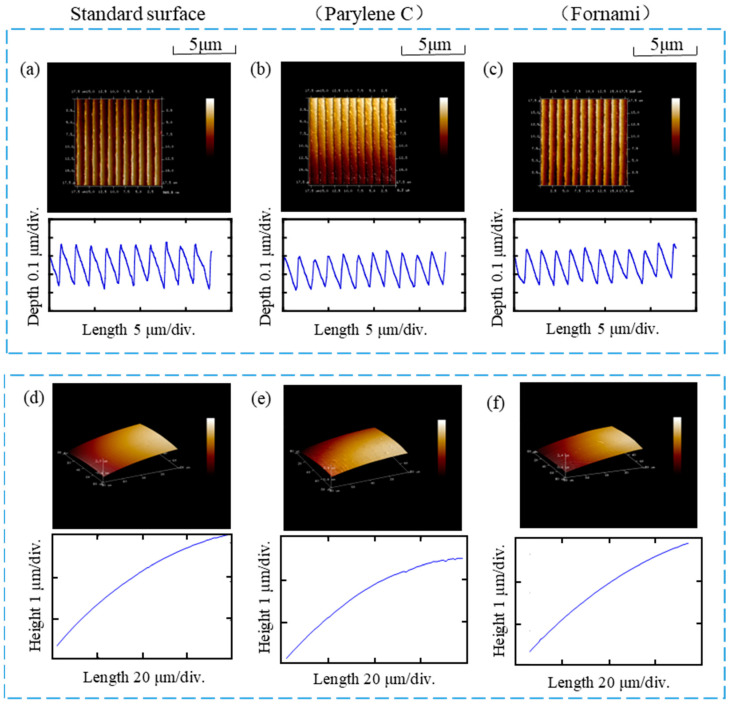
Characterization of the finished microlens array grating: (**a**) the standard grating surface with its depth and period; (**b**) the grating surface with its depth and period with Parylene is used as anti-adhesive coatings in the fabrication process; (**c**) the grating surface with its depth and period when Fluorine Nano is used as anti-adhesive coatings in the fabrication process; (**d**) the standard microlens surface; (**e**) the microlens when Parylene is used as anti-adhesive coatings in the fabrication process; (**f**) the microlens when Fluorine Nano is used as anti-adhesive coatings in the fabrication process.

The prepared samples were investigated for surface morphology of the grating and microlens array using Bruker’s Innova AFM at A. The probe lightly touched the grating surface, moving approximately 10 grating cycles away, and performed five scans on the same plane. The obtained results were flattened, and a cross-section was taken perpendicular to the grating lines to measure the cross-sectional characteristics of the grating groove shape, which provided data on grating height and period curves. These results are illustrated in Figure 5a–c, with corresponding morphological parameters presented in Table 4. Analysis of the test results indicates an average depth deviation of 2.2% and an average width deviation of 0.1% compared to the pristine grating template, aligning with expectations.

The lens surface was also measured using AFM to obtain its morphological information. The measurement results were flattened, and the surface’s radius of curvature was fitted by selecting three random points on the surface. This process was repeated five times, and the average values were calculated to obtain the microlens height curve and determine its radius of curvature. Since the microlens surface is replicated using an inverted mold, which is associated with the anti-adhesive coating, the manufactured products with two different coatings, P (Parylene C) and F (Fluorine nano), were tested separately. The measurement results are depicted in Figure 5d–f, with the microlens morphology parameters presented in Table 5. The average radius of curvature for the microlens template was 1.768, while the microlens made with the P coating and F coating had average radii of curvature of 1.755 and 1.806, respectively. This represents a deviation of 0.7% for the former and 2.1% for the latter compared to the radius of curvature of the original microlens surface. It is apparent that the samples prepared with the P coating exhibit better quality and were therefore utilized in subsequent microlens–grating spectrometer experiments.

## 4. Verification

### 4.1. Characterization of Spectrometer (Calibration)

**Figure 6:**
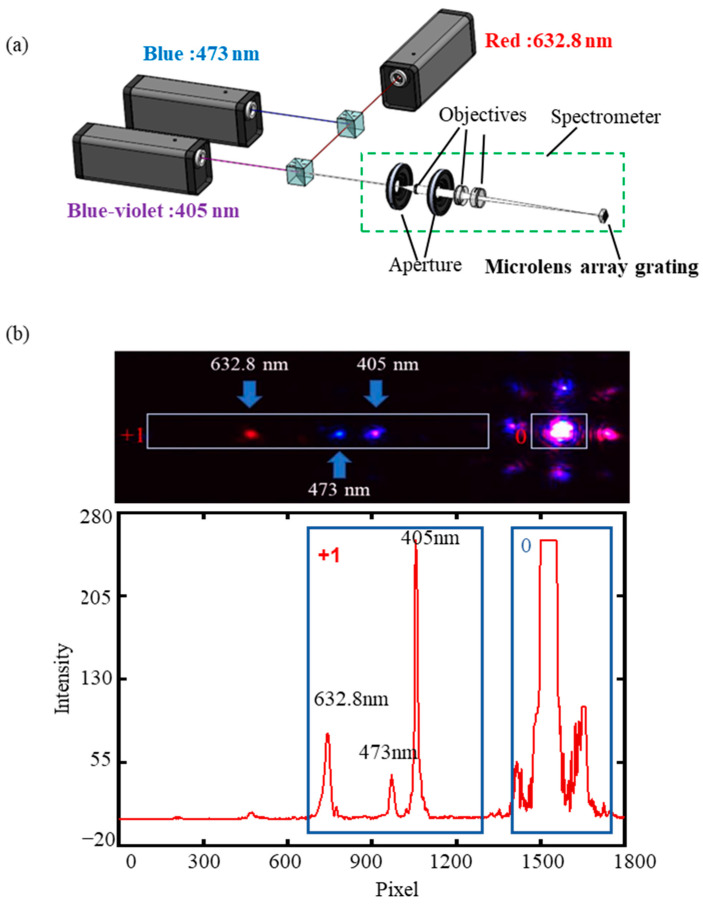
Spectral analysis ability testing system and results: (**a**) schematic diagram of the miniature spectrometer system; (**b**) results in a single microlens surface element.

An optical setup is shown in Figure 6a. The experimental system consists of the synthetic laser light source, three-piece objectives, CMOS detector (MV-CA060-10GC by HIKVISON), and computer. Laser beams of wavelengths of 632.8 nm (red), 473 nm (blue), and 405 nm (blue–violet) were combined and introduced to the fiber port. The light emitted from the fiber optic port was dispersive. After reducing the dispersion angle by a three-piece objective system, the light beam was focused and dispersed by the fabricated component. Light was imaged and recorded on the CMOS detector surface. Finally, the images were shown and analyzed by software on the computer. The image obtained by the CMOS detector is shown in Figure 6b. Since the grating is a diffractive element, the light spots appearing on the image are diffracted spots at various levels. The brightest spot is zero level spot and the other three spots are the first level diffraction spots. The centers of these spots are connected as the sampling axis. The light intensity on the sampling axis was recorded and is shown in Figure 6b. The intensity of zero level spot was higher than the intensity of the first level diffraction spot. According to the first level diffraction spots, the light beams with different wavelengths were distributed at different positions and recorded by pixels. The larger the wavelength is, the farther the center of the spot is from the zero-level spot. The mass center method was used to obtain the position of the spot center of first level diffraction spot. The real calculated method is shown in Equation (3) where $P_{CM}$ is the focus pixel, $P_{n}$ is the pixel number, $I\left(P_{n}\right)$ is light intensity at pixel number $P_{n}$.

(3) $$P_{CM}=\frac{\sum P_{n}\cdot I\left(P_{n}\right)}{\sum I\left(P_{n}\right)}$$

Four surface elements were chosen at random and the images were recorded in order to test the performance of different surface elements. The results are shown in Table 6. The standard deviations of pixel positions were 0.81, 0.82, 0.95 for the different laser colors. The good consistency of different spectrometer units was verified.

### 4.2. Application in Chromatic Confocal System

The feasibility of microlens array grating as the core component of the spectrometer has been verified in the previous section. In this section, the performance of the microlens array-grating spectrometer in a spectral confocal system is presented.

In the optical signals containing position information acquired by the chromatic confocal probe, the focused wavelength has the highest intensity, so it can be regarded as monochromatic light. The resolution of the chromatic confocal sensor depends on the wavelength resolution of the spectrometer to some extent.

**Figure 7:**
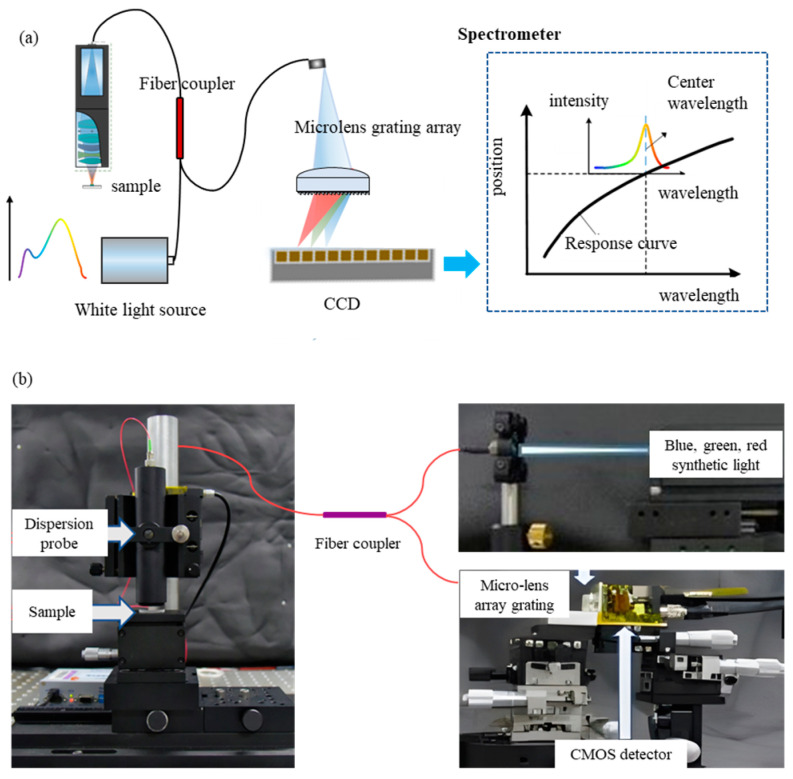
Application testing of microlens array grating spectrometer in spectral confocal measurement system: (**a**) schematic of the chromatic confocal system; (**b**) picture of the chromatic confocal system.

As Figure 7a shows, the chromatic confocal sensor consists of the polychromatic light source, dispersive objectives, fiber coupler, and the spectrometer. Microlens array grating was used as the core element of the spectrometer in this system. In order to fit the small numerical aperture of microlens array grating, a white laser source, which was synthesized by three wavelengths, was used and compared with LED. The laser source has higher energy to make sure the optical signal can be noted by CMOS after the energy loss caused by the light path.

**Figure 8:**
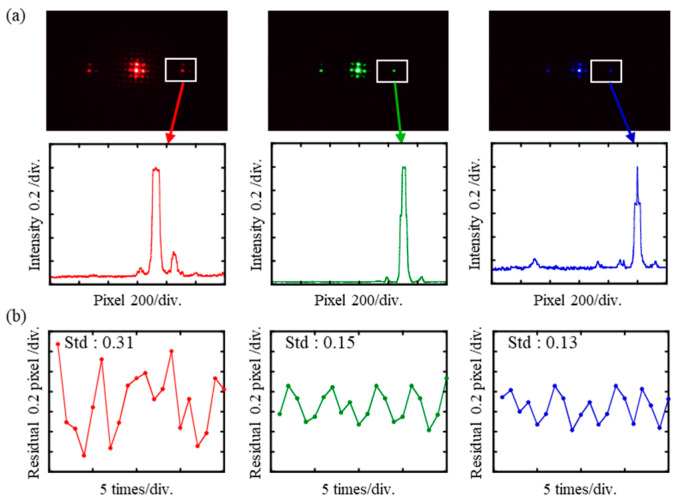
Experimental results: (**a**) optical signal detected by CMOS and its intensity (**b**) residual error.

A z-stage was used to make the calibration experiments. Due to the principle of chromatic confocal sensors, different positions of samples correspond to different wavelengths of reflected light. As shown in Figure 8, the images can be recorded by controlling the z-stage. When the sample was in different positions, the color of the spot obtained was different. The curve of the pixel–wavelength relationship can be obtained by calibration according to the previous section. In the chromatic confocal system, there was a correspondence between the sample position and the spot position in the whole system since there is a functional relationship between the position and the focused wavelength. In Figure 8a, the light spot positions of three measurements are P1, P2, and P3, respectively. The displacement stage was controlled to reach the corresponding position after recording the position of the z-stage and the pixel position at the center of the spot for each measurement. Figure 8 shows the results of the experiments, which were carried out 20 times. The standard deviations of pixel positions were 0.31, 0.15, and 0.13, respectively. In conclusion, the spectrometer showed good stability in the chromatic confocal system.

## 5. Conclusions

A microlens array gating (MLAG), which has the functions of focusing and dispersion, is proposed for the miniature multi-channel spectrometer. The single microlens with a bin size of 0.22 mm and focal length of 4 mm was arrayed on one side of the component with a dimension of 10 mm × 10 mm × 4 mm, namely, there are more than 2000 microlenses on the surface. The other side of the component was a blazed grating with a density of 600 lines/mm. The fabrication of the microlens array grating was achieved by soft lithography, which consisted of four steps: PDMS preparation, primary replication process, anti-adhesive treatment process, and secondary replication process. The quality of the fabricated MLAG was confirmed through characterization using AFM microscopy. To verify the performance of the developed miniature multi-channel spectrometer utilizing the proposed MLAG, the centroid position repeatability of the diffractive light spot was detected by the centroid method. The results demonstrate that the centroid position repeatability of the diffractive light spot was kept within 1 pixel, and the performance of different microlens grating panels was consistent. The experiments involving spectral confocal measurement were carried out to verify the application of a miniature multi-channel spectrometer with an MLAG. The results reveal that the repeatability of the spot centroid position was within 0.5 pixels at the three typical positions within the corresponding 400 μm measuring range, which is expected to bring a measurement resolution of 0.5 μm for the spectral confocal system.

## Figures and Tables

**Table 1:** Resolution of the spectrometer at different wavelengths.

Wavelength/nm	Resolution/nm
400	13
450	12
470	11
550	9
600	9
625	7
650	6
675	8
700	9

**Table 2:** Parameters of primary replication mold.

Parameter	Upper Mold	Bottom Mold
Side length	40 mm	40 mm
Thickness	5 mm	4 mm
Groove shape	Circle	Circle
Groove diameter	12.7 mm	16.2 mm
Groove depth	5 mm	2 mm
Positioning hole diameter	4 mm	3 mm

**Table 3:** Parameters of secondary replication mold.

Parameter	Upper Mold	Middle Mold	Bottom Mold
Side length	40 mm	40 mm	40 mm
Thickness	4 mm	4 mm	10 mm
Groove shape	Circle	square	Square
Groove diameter	16.2 mm	10 mm	13.7 mm
Groove depth	2 mm	4 mm	6 mm
Positioning hole diameter	3 mm	2 mm	2 mm

**Table 4:** Grating surface morphology parameters.

Samples	Grating in MLGA	Grating
Width 8 cycles/μm	13.093	13.115
Average width/μm	1.637	1.639
Width deviation	0.10%	-
Average depth/nm	183.4	187.5
Depth deviation	2.20%	-

**Table 5:** Micro-lens surface morphology parameters.

Samples	Micro-Lens in MLGA		Micro-Lens
Coating material	P	F	-
Curvature/mm^−1^	1/1.755	1/1.806	1/1.768
Deviation	0.70%	2.10%	-

**Table 6:** Results in a single microlens surface element.

No.	632.8 nm	473 nm	405 nm
1	773	541	458
2	772	541	457
3	771	542	456
4	772	540	456
Std (pixel)	0.81	0.82	0.95
Sta (nm)	0.48	0.64	0.81
Simulation (nm)		Around 0.5	
